# Global Substrate Profiling of Proteases in Human Neutrophil Extracellular Traps Reveals Consensus Motif Predominantly Contributed by Elastase

**DOI:** 10.1371/journal.pone.0075141

**Published:** 2013-09-20

**Authors:** Anthony J. O’Donoghue, Ye Jin, Giselle M. Knudsen, Natascha C. Perera, Dieter E. Jenne, John E. Murphy, Charles S. Craik, Terry W. Hermiston

**Affiliations:** 1 US Innovation Center, Bayer Healthcare Pharmaceuticals, San Francisco, California, United States of America; 2 Department of Pharmaceutical Chemistry, University of California San Francisco (UCSF), San Francisco, California, United States of America; 3 Comprehensive Pneumology Center, Institute of Lung Biology and Disease (ILBD), Helmholtz Zentrum München, Member of the German Center for Lung Research, Munich, Germany; 4 Max-Planck-Institute of Neurobiology, Planegg-Martinsried, Germany; University of Toronto, Canada

## Abstract

Neutrophil extracellular traps (NETs) consist of antimicrobial molecules embedded in a web of extracellular DNA. Formation of NETs is considered to be a defense mechanism utilized by neutrophils to ensnare and kill invading pathogens, and has been recently termed NETosis. Neutrophils can be stimulated to undergo NETosis ex vivo, and are predicted to contain high levels of serine proteases, such as neutrophil elastase (NE), cathepsin G (CG) and proteinase 3 (PR3). Serine proteases are important effectors of neutrophil-mediated immunity, which function directly by degrading pathogenic virulent factors and indirectly via proteolytic activation or deactivation of cytokines, chemokines and receptors. In this study, we utilized a diverse and unbiased peptide library to detect and profile protease activity associated with NETs induced by phorbol-12-myristate-13-acetate (PMA). We obtained a “proteolytic signature” from NETs derived from healthy donor neutrophils and used proteomics to assist in the identification of the source of this proteolytic activity. In addition, we profiled each neutrophil serine protease and included the newly identified enzyme, neutrophil serine protease 4 (NSP4). Each enzyme had overlapping yet distinct endopeptidase activities and often cleaved at unique sites within the same peptide substrate. The dominant proteolytic activity in NETs was attributed to NE; however, cleavage sites corresponding to CG and PR3 activity were evident. When NE was immunodepleted, the remaining activity was attributed to CG and to a lesser extent PR3 and NSP4. Our results suggest that blocking NE activity would abrogate the major protease activity associated with NETs. In addition, the newly identified substrate specificity signatures will guide the design of more specific probes and inhibitors that target NET-associated proteases.

## Introduction

Neutrophils are the most abundant leukocytes in plasma. They are the first cells recruited to injury sites in response to pathogen invasion, and they act as the first line of innate immune defense. Neutrophils have traditionally been considered effector cells for the inflammatory response and acute immunity, functioning through intracellular phagocytosis, and using lytic proteases, reactive oxygen species and microbicidal proteins to attack infective agents. Recent studies have shown that neutrophils also have the capacity to regulate the immune response by expressing cytokines, chemokines, Fc receptors and complement components for signaling with other immune cells, such as dendritic cells, B cells and T cells [1].

Proteases are important effectors of neutrophils. They not only contribute directly to microbicidal activity but also function in the proteolytic processing of chemokines, cytokines and receptors [2,3]. This modulatory activity is exemplified by the caspase-independent activation of IL-1β and IL-18 by NE, PR3 and CG [4,5,6] or the conversion of anti-inflammatory progranulin to pro-inflammatory granulin by NE and PR3 [7]. Furthermore, NE has been shown to couple neutrophil-mediated inflammation with the coagulation pathway by cleaving tissue factor pathway inhibitor on Neutrophil Extracellular Traps (NETs).

NETs are released by stimulated neutrophils in a specific form of cell death called NETosis. NETosis is hypothesized to represent a new mechanism of innate immunity mediated by neutrophils in response to pathogen invasion [8,9]. It is characterized by the formation of NETs, networks made of decondensed chromatin and anti-microbial proteins and peptides. NETosis represents a new paradigm of cell death that is distinct from apoptosis and necrosis in many aspects. No nuclear fragmentation or membrane blebbing is observed, and activation is independent of caspase activity, although NADPH oxidase is required [10]. NETosis also involves activities of NE and myeloperoxidase [11], histone citrullination by peptidylarginine deiminase 4 [12] and activation of the Raf-MEK-ERK pathway [13]. It has been hypothesized that the primary function of NETs is to trap and kill pathogens. In addition, it also provides a matrix for establishing high local concentrations of effectors and mediators for the ensuing innate and adaptive immune responses.

Stimulation of neutrophils with PMA causes the release of each of the serine proteases NE, PR, CG [14] and NSP4 [15] and the metalloproteases, MMP-8 and MMP-9 [16]. However, only NE, PR and CG have been found to be associated with the resulting NET [14]. To characterize NET-associated proteolytic activities in an unbiased manner, proteins trapped in NETs were released and assayed with the Multiplex Substrate Profiling by Mass Spectrometry (MSP-MS) method [17]. This method utilizes a library of 124 highly diversified peptides in a multiplex assay with tandem liquid chromatography-mass spectrometry for detection of cleavage sites. Using the MSP-MS assay, a proteolytic signature was uncovered for each donor sample and compared to the signatures generated for each neutrophil serine protease. By deconvolution of the NETs’ associated protease activities we determined that the major activity was attributed to NE. Immunodepletion of NE activity revealed important contributions from PR3 and to a lesser extent CG and NSP4. Identifying the substrate specificity and the contribution of each NET-associated protease to overall NET-associated activity could lead to the development of therapeutics to alleviate pathological NETosis in acute and chronic immune diseases.

## Results

### Induction of NETs with enriched protease activity

**Figure 1:**
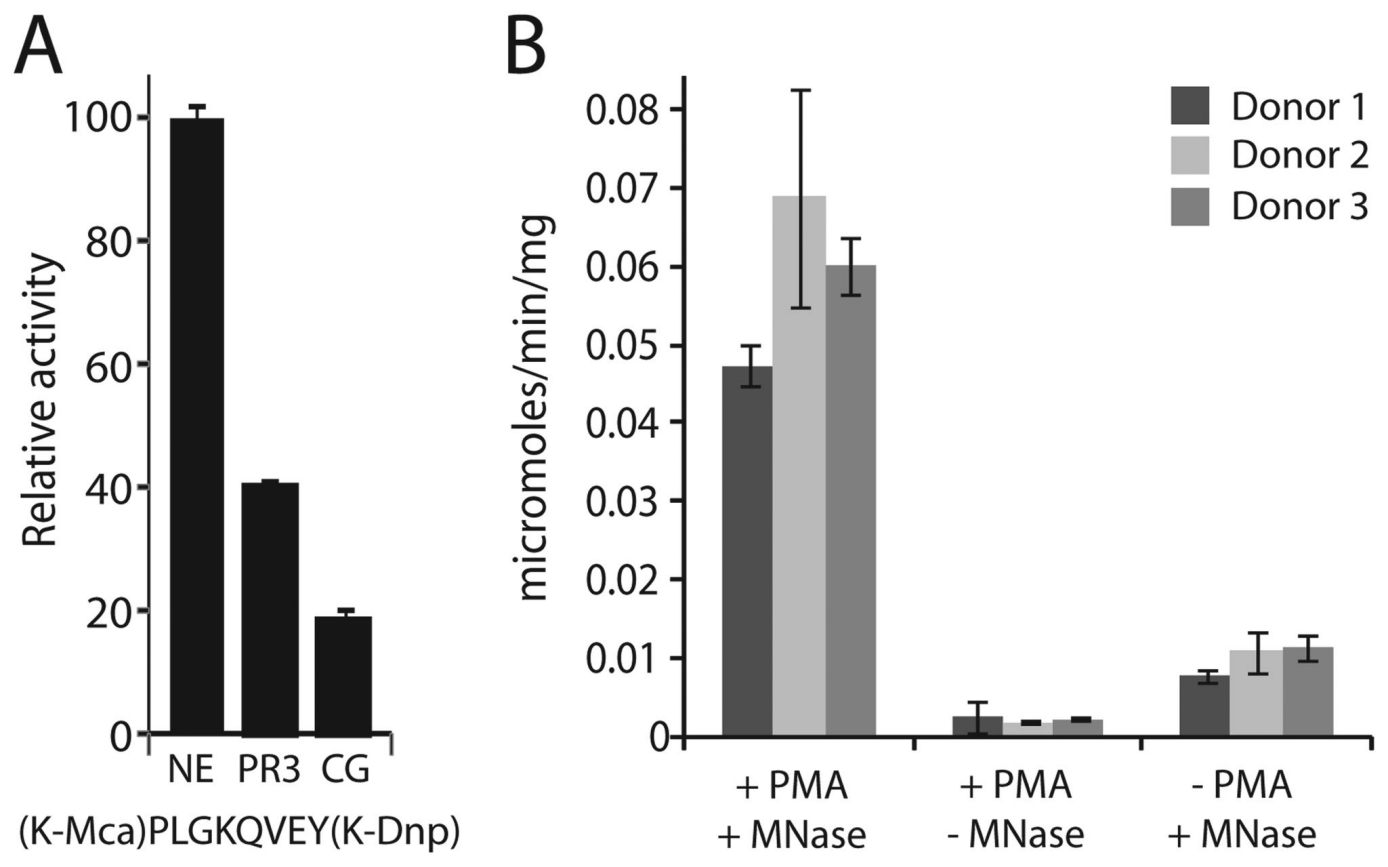
Monitoring of proteolytic activity in neutrophil extracellular traps. **A**. Identification of an internally quenched fluorescent substrate that is hydrolyzed by NE, CG and PR3. **B**. Extracellular proteolytic activity was analyzed from 3 donor neutrophils (Donor 1, dark grey; Donor 2, black; Donor 3, light grey) following treatment with PMA, MNase or a combination of both. Proteolytic activity was measured using (K-Amc) PLGKQVEY(K-Dnp).

NE, PR3 and CG were previously identified in NETs and together were estimated to make up ~9% of the total protein associated with the NETs [14]. To estimate proteolytic activity in PMA-induced NETs generated from healthy donor neutrophils we screened a set of internally quenched fluorescent peptides and identified a substrate that was readily cleaved by all three enzymes (Figure 1A, Figure S1). This substrate, K(Mca)-PLGKQVEY-K(Dnp), was previously used to assay a glutamic acid protease secreted from a fungus [18]. Using this probe, the proteolytic activity released from NETs derived from PMA and Micrococcal Nuclease (MNase) treated neutrophils ranged from 0.047 to 0.068 µmoles/min/mg. For individual donors this activity was approximately five-fold greater than control samples that lack PMA treatment, and twenty- to forty-fold greater than control samples that lacked MNase treatment (Figure 1B). These assays confirmed that activated neutrophils produce NETs that are enriched with proteases that can be released upon nuclease treatment.

### Comparison of substrate signatures of NET associated proteases from donor neutrophils

**Figure 2:**
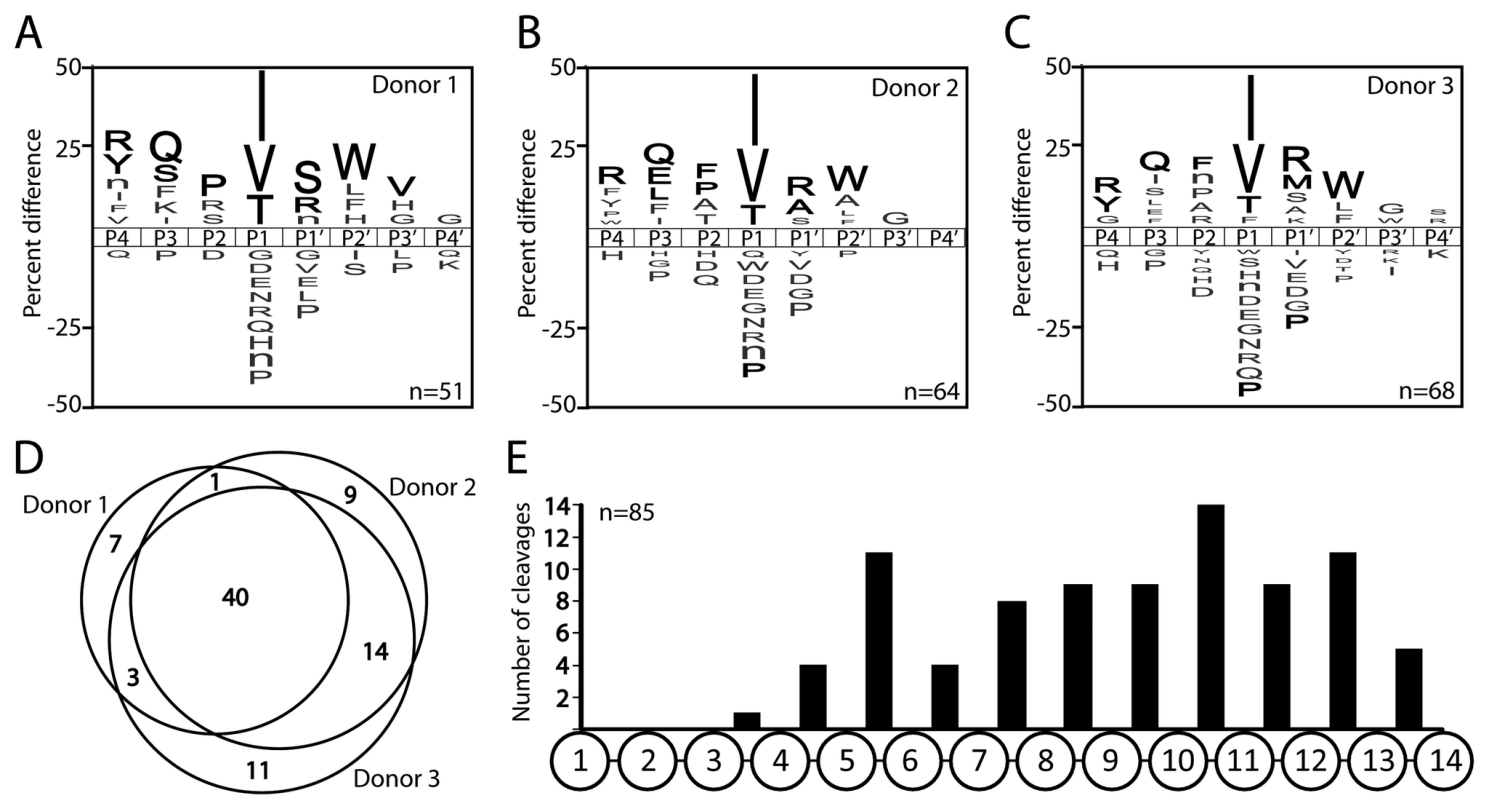
Determination of the proteolytic signatures in NETs. **A**-**C**. IceLogos representing the P4 to P4′ sites for NETs isolated from three donor samples. Amino acids that are most frequently observed (above axis) and least frequently observed (below axis) are illustrated. The number of cleavage sites used to make each iceLogo are listed in the bottom right-hand corner. Residues that are highlighted in black text are significantly (p = 0.05) enriched relative to the frequency that these same amino acids are found in the peptide library (5.2 +/- 0.5%). The amino acid ‘n’ corresponds to norleucine. **D**. Determination of the number of cleavage sites that are common and unique to each donor sample. **E**. Positional frequency of all donor-derived cleavage sites within the tetradecapeptides (n=85).

We employed MSP-MS to generate a substrate signature of proteases associated with NETs from PMA activated neutrophils and compared the substrate specificity for each donor sample (Figure 2A-C). Each donor sample contained proteases with a distinct preference for isoleucine, valine and threonine in the P1 position while arginine, glutamine and tryptophan were significantly enriched (p ≤ 0.05) at P4, P3 and P2′ positions, respectively. Furthermore, cleavage was rarely observed on the C-terminal side of amino acids with charged side chains with the exception of lysine, while proline is not well tolerated in either the S3, S1 or S1′ pockets of these proteases. When donors were directly compared, 40 shared cleavage sites were identified and each donor sample had between 7 and 11 unique cleaved bonds (Figure 2D). When the location of each cleavage site was analyzed, no hydrolysis was evident near the N-termini of the tetradecapeptides, indicating that these neutrophil derived enzymes lacked aminopeptidase specificity (Figure 2E). However, cleavage readily occurred near the C-termini, indicating the presence of carboxypeptidases or endoproteases that have little specificity at the non-prime side of the scissile bond.

### Identification of NET associated proteins

To identify the full complement of proteins embedded in the NETs, protein preparations from the same NETosis-induced neutrophils described above were subjected to proteomic analysis to evaluate the protein composition of neutrophils after PMA and MNase treatment (Table 1). Using mass spectrometry, 29 proteins were identified in PMA- and MNase-treated neutrophil samples, however only NE, alpha-enolase and Histones H2A and H3 were found to be specifically enriched relative to the control samples. Eleven proteins were reproducibly identified in all three PMA- and MNase-treated donor samples, which included NE and the inactive serine protease family member, azurocidin. Surprisingly, while our enzymatic studies indicated an enrichment of proteolytic activity in NETs from PMA- and MNase-treated neutrophils relative to the control samples, there was little or no enrichment of proteases in the same samples when analyzed by mass spectrometry-based proteomics. CG was found in one donor sample while PR3, NSP4 and MMP-8 were never observed. Two peptides corresponding to MMP-9 were observed in a MNase only treated sample.

**Table 1:** NET associated proteins identified by LC-MS/MS from three healthy donors.

**UniProt Accession**	**Unique Peptides**	**Mean Peptide Count (n**)	**Number Unique**	**Peptide Count**	**Number Unique**	**Peptide Count**	**Protein MW**	**Protein Name**
B4E335	43	17.33 (1)	97	173	73	141	39226.3	Actin, beta
P05164	99	97.33 (3)	27	43	35	47	83869.4	Myeloperoxidase
A8K9U8	71	41.67 (2)	80	128	43	65	78338.9	Lactoferrin
P08246	59	68.33 (3)	-	-	36	64	28518.3	Neutrophil elastase
B4DLA9	43	42 (1)	14	24	21	47	14841.5	Histone H2B
B2R4P9	43	54.33 (3)	-	-	-	-	15328	Histone H3
A3KPC7	38	39.33 (2)	-	-	-	-	13906.4	Histone H2A
P20160	33	32.67 (3)	10	13	12	13	26885.9	Azurocidin
B2R4R0	30	51 (3)	8	9	20	41	11367.4	Histone H4
P35579	-	-	23	30			226534.2	Myosin-9
B3KSI4	8	2.67 (1)	8	11	24	31	58982.1	Transketolase-like
P06733	14	7.33 (3)	-	-	20	32	47169.4	Alpha-enolase
A3R0T8	11	7 (3)	18	25	7	12	21865.4	Histone 1, H1e
B2R4C5	17	6.33 (1)	21	23	18	23	16537.2	Lysozyme
P08311	15	6 (1)	14	20	10	13	28837.5	Cathepsin G
B2R4M6	3	1 (1)	14	24	16	28	13210.1	Protein S100-A9
P59665	14	25 (3)	16	59	13	37	10201.1	Neutrophil defensin 1
A4UCT1	10	4.67 (3)	11	13	14	20	17303.1	Glyceraldehyde-3-P dehydrogenase
B2R829	-	-	20	27	-	-	45850.5	MNDA-like
A2A418	4	1.33 (2)	13	14	13	17	80641.3	Gelsolin
C5HZ13	-	-	-	-	12	21	16453	Charcot-Leyden crystal protein
B4DE36	2	1 (2)	-	-	8	10	60186.3	Glucose-6-phosphate isomerase
P07737	3	3 (2)	3	3	7	10	15054.4	Profilin-1
P31146	5	1.67 (1)	-	-	11	22	51026.7	Coronin-1A
A8K4W6	-	-	7	8	9	11	44615.1	Phosphoglycerate kinase
B4DNK4	-	-	6	7	-	-	49898.2	Pyruvate kinase
P80723	-	-	6	6	3	3	22693.6	Brain acid soluble protein 1
B0YJC4	-	-	-	-	10	16	49653.8	Vimentin
B4E112	-	-	3	6	4	7	12459.7	Cofilin-1-like
A2VCK8	4	4 (2)	8	25	5	22	5052.7	Thymosin beta 4, X-linked
P12724	-	-	6	7	-	-	18385.5	Eosinophil cationic protein
P05109	5	2.67 (3)	3	5	7	9	10834.6	Protein S100-A8
A8K220	-	-	3	4	4	7	18012.7	Peptidyl-prolyl cis-trans isomerase
A8MX94	-	-	-	-	3	3	19480.7	Glutathione S-transferase P
F2Z393	1	0.33 (1)	-	-	4	4	35329.2	Transaldolase
B4DJI2	-	-	4	6	1	2	56853.5	Granulins-like
A8K2Y9	-	-	-	-	5	6	53140.5	6-P gluconate dehydrogenase
P49913	-	-	4	4	-	-	19301.6	Cathelicidin antimicrobial peptide
O60234	1	0.33 (1)	3	3	-	-	16801.5	Glia maturation factor gamma
D6R9A6	1	0.67 (1)	6	14	-	-	15403.9	High mobility group box 2
A8K2L4	-	-	3	4	1	1	37247.9	LSP1-like
P80511	2	1 (1)	-	-	-	-	10575.1	Protein S100-A12
B3KUI1	1	1.33 (3)	3	4	3	4	25043.3	Plastin-2-like
B5BU38	-	-	-	-	3	4	38680.6	Annexin
A7XZE4	-	-	2	3	3	3	33026.2	Beta tropomyosin isoform
Q5T123	3	1.67(2)	2	3	-	-	9380.6	SH3BGRL3
P05204	-	-	3	8	2	11	9392.7	HMGN2
B0QZK8	-	-	2	2	-	-	13683.9	HP1BP3
B7Z507	-	-	-	-	2	2	71554.8	MMP9-like
Q68D08	-	-	-	-	2	2	36750.3	DKFZp686B04128

The number of unique peptide counts corresponds to the total number of unique peptide sequences that identify a protein entry. The peptide count is the number of peptide spectra observations for a given protein, either reported as the mean across three replicate experiments or individually for the control experiments.

### MSP-MS profiling of NE and comparison to NETs

**Figure 3:**
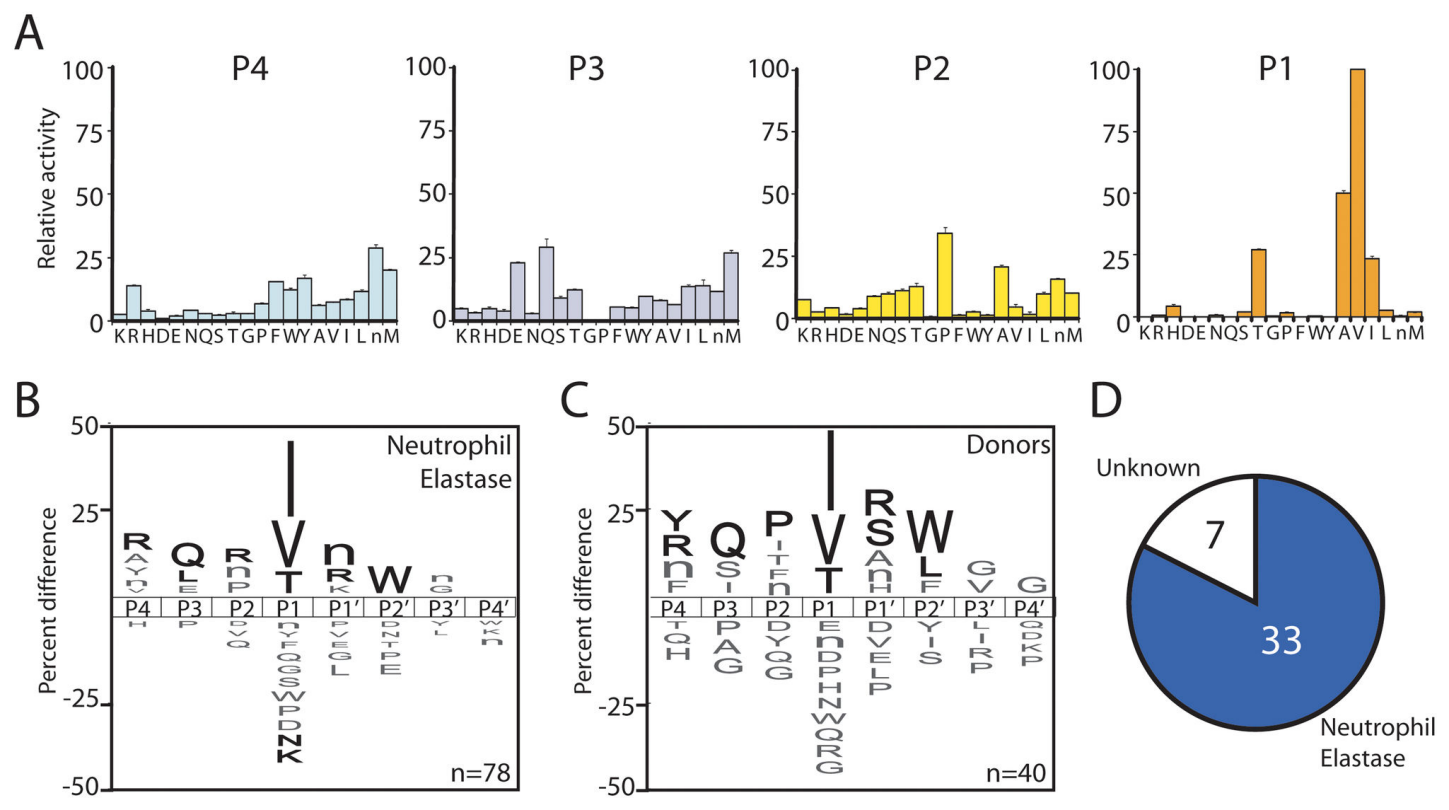
Substrate specificity profiling of human Neutrophil Elastase A. Positional scanning of the P4 to P1 subsites of NE using the PS-SCL assay. **B** An iceLogo illustrating amino acids that are most frequently (above axis) and least frequently (below axis) observed in the P4 to P4′ sites of NE. Residues that are highlighted in black are significantly (p = 0.05) enriched or de-enriched in the subsites relative to the frequency that these same amino acids are found in the peptide library (5.2 +/- 0.5%). **C**. A representative “donor signature” consisting of 40 cleavage sites that are common to the three donors. **D**. A pie chart representing the 40 cleavage sites that are common to the donor samples. 33 of these sites are also hydrolyzed by NE.

Based on our proteomic data, the majority of proteolytic activity in NETs was predicted to be derived from NE. To test this prediction, the substrate specificity of purified NE was profiled using both MSP-MS and the more established positional scanning synthetic combinatorial library (PS-SCL) assay [19]. This enzyme was isolated from human neutrophils and was found to be free of other neutrophil or serum proteases using mass spectrometry based proteomic analysis (data not shown). In the PS-SCL assay the P1 site of NE had a distinct preference for valine, alanine, threonine and isoleucine. In addition, proline was preferred in P2, glutamine, glutamic acid and methionine in P3 and norleucine in P4 (Figure 3A). Using the MSP-MS assay, NE had a preference for isoleucine, valine and threonine in the P1 position and had low tolerance for asparagine, lysine and most other amino acids with charged side chains. In addition arginine, glutamine, norleucine and tryptophan were significantly enriched (p ≤ 0.05) at the P4, P3, P1’ and P2′ positions, respectively. A Pearson correlation was performed on the NE profiles from the MSP-MS and PS-SCL methods which showed that each non-prime subsite had strong positive correlation with a score of ≥0.4 on a scale from -1.0 to 1.0 (Table 2). This validated the MSP-MS assay as a method for profiling the substrate specificity of NE and other serine neutrophil serine proteases.

**Table 2:** Comparison of NE substrate specificity using MSP-MS with NET Donors and NE using PS-SCL.

	**P4**	**P3**	**P3**	**P1**	**P1′**	**P2′**	**P3′**	**P4′**
NE (PS-SCL)	0.50	0.82	0.41	0.65	-	-	-	-
Donors	0.63	0.85	0.70	0.95	0.69	0.79	0.58	0.34

Pearson correlation values of <0.4 indicate weak or no correlation while values ≥0.4 and ≥0.7 represent strong and very strong correlation, respectively.

To generate a representative “donor signature”, the 40 cleavages observed in all three donor samples were aggregated into a single motif. An advantage of using the MSP-MS assay over the PS-SCL assay for profiling biological samples is that specific cleavage of peptide substrates can be directly linked to a protease. The cleavage sites that were common to all three donors were directly compared with the substrate profile of purified NE (Figure 3C) and correlated strongly between P4 and P3′ (Table 2). In addition we determined that 33 of the cleaved bonds that occur in donor samples could also be hydrolyzed by NE (Figure 3C) which provided evidence that the major proteolytic activity in NETs is derived from NE. However, seven of the cleavage sites could not be directly linked to NE activity, therefore additional proteases were active on NETs.

### MSP-MS profiling of CG, PR3 and NSP4 and comparison to NETs

**Figure 4:**
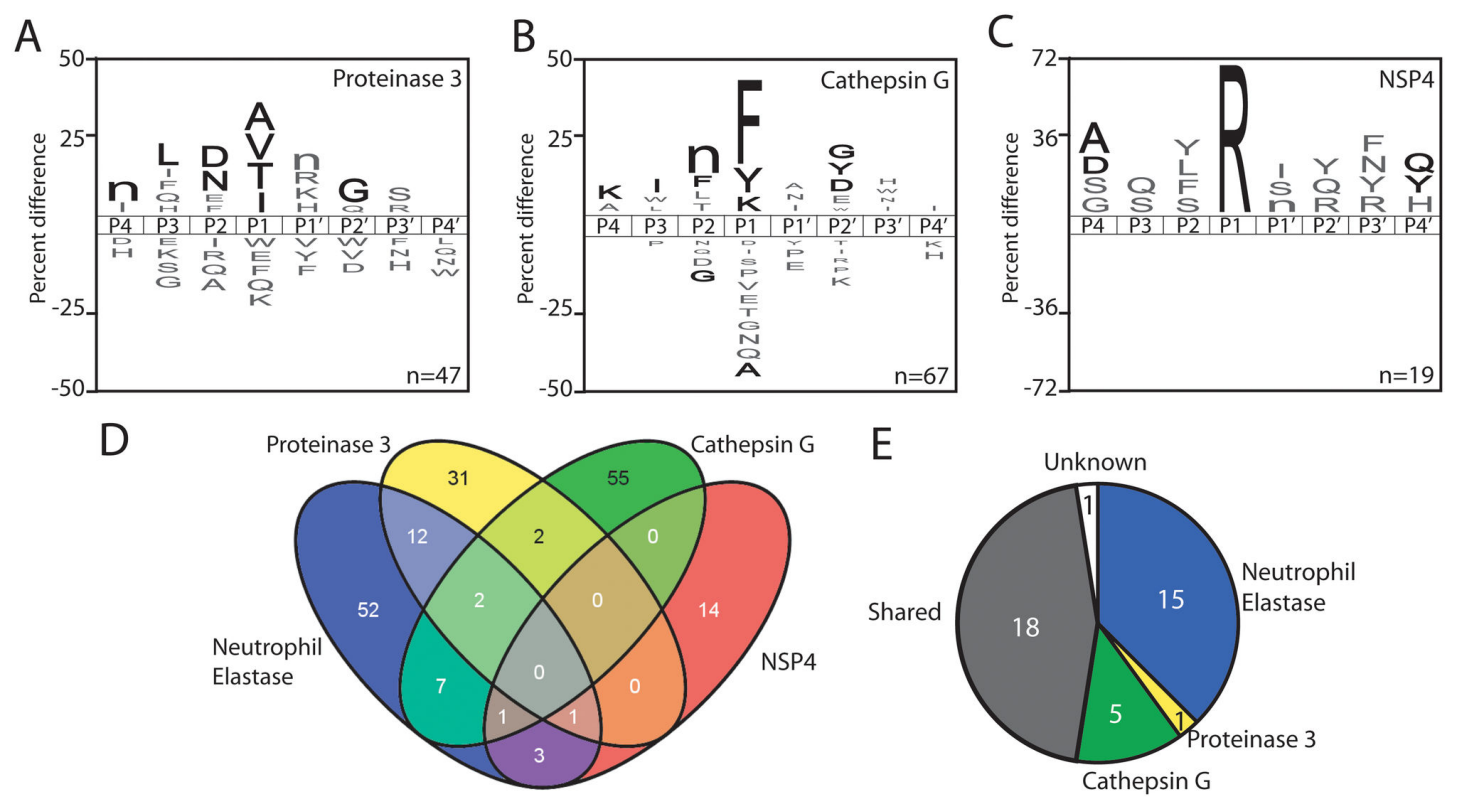
Determination of proteolytic signature of PR3, CG and NSP4. **A**-**C**. IceLogos representing the P4 to P4′ sites for PR3, CG and NSP4. Amino acids that are most frequently observed (above axis) and least frequently observed (below axis) are illustrated. The number of cleavage sites used to make each iceLogo are listed in the bottom right-hand corner. Residues that are highlighted in black text are significantly (p ≤ 0.05) enriched relative to the frequency that these same amino acids are found in the peptide library (5.2 +/- 0.5%). The amino acid ‘n’ corresponds to norleucine. **D**. A 4-way venn diagram illustrating the number of unique and overlapping peptide bonds that are cleaved by each neutrophil serine protease. **E**. A pie chart representing the 40 cleavage sites that are common to the donor samples. ‘Shared’ corresponds to cleavage sites that are derived from more than one neutrophil serine protease.

CG, PR3 and the newly identified neutrophil serine proteases NSP4 have been shown to be released from PMA activated neutrophils [14,15], therefore these enzymes were profiled using MSP-MS to identify the source of the unknown proteolytic activity in NETs. In the P1 positions, PR3 had approximately equal preference for alanine, valine, threonine and isoleucine, CG favored phenylalanine over tyrosine and lysine, while NSP4 had a strict preference for arginine (Figure 4A-C). Outside of the P1 subsite, PR3 showed selectivity for aspartic acid and asparagine at P2, and norleucine, leucine and glycine at P4, P3 and P2′, respectively while CG had a preference for norleucine at P2. In contrast to the other neutrophil serine proteases, NSP4 specificity was so selective for P1 arginine residues that only 19 bonds were cleaved in the library after 20 hours incubation. In order to further characterize non-prime side specificity, each enzyme was assayed using the PS-SCL method and the P4 to P1 site preferences were identified (Figure S2).

Using the MSP-MS datasets, a Venn diagram was generated to highlight the unique and shared cleavage sites for each of the neutrophil serine proteases. Interestingly, for a family of enzymes that share high sequence identity, they have each evolved to cleave a defined set of unique peptide bonds. When compared to the unknown cleavage sites in NETs, we determined that 5 of the 7 sites were matched to CG while one site was matched to PR3 activity. With the knowledge that CG and PR3 are active in NETs we were now only able to directly assign 18 of the cleavage sites to NE as many of the sites can be cleaved by more than one neutrophil serine protease (Figure 4E). A single unidentified cleavage site occurred between valine and isoleucine in a peptide containing the sequence PHWQRVIFFRLNTP. Although not observed in the individual enzyme assays, cleavage at this P1 valine site is likely to be the result of NE, CG or both.

### Immunodepletion of NE from NETs

**Figure 5:**
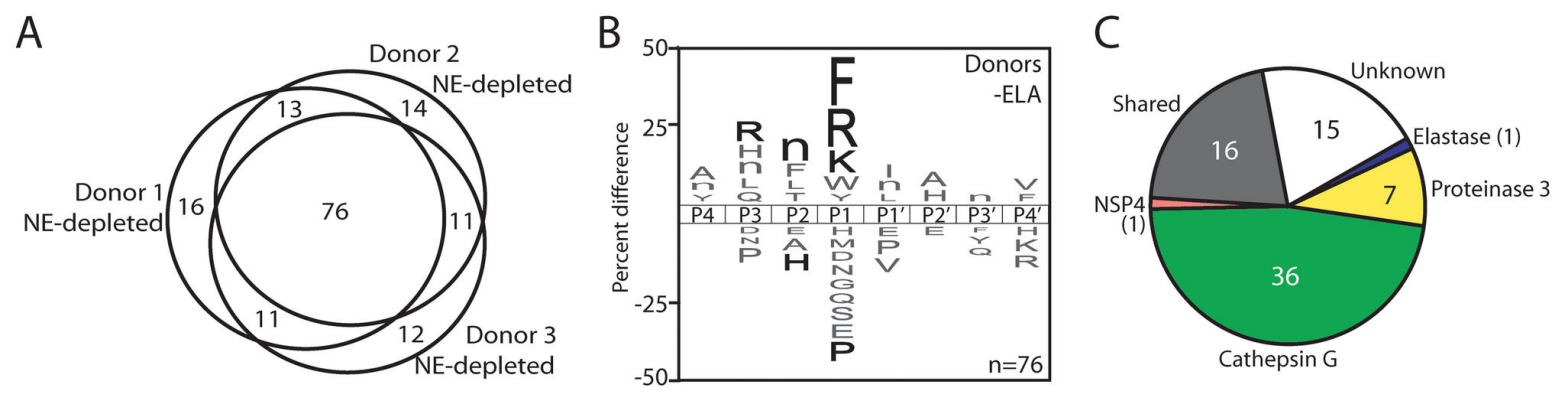
Determination of the proteolytic signatures in NE-depleted NETs. **A**. After immunodepletion of NE from donor NET samples, the MSP-MS assay was performed at 15-fold higher total protein concentration. A total of 153 cleaved bonds were identified in the 3 donor samples and 76 cleaved bonds were common to each donor. **B**. An iceLogo illustrating the proteolytic signature of cleavage sites that are common to each NE-depleted donor. **C**. A pie chart representing the 76 cleavage sites that are common to the donor samples. ‘Shared’ corresponds to cleavage sites that are derived from more than one neutrophil serine protease.

In order to confirm that NE was the major proteolytic activity in NETs, we selectively removed the enzyme by immunodepletion, and assayed the remaining proteases in the mixture. On this occasion, we were able to increase the total amount of NE-depleted donor protein in the assay by 15-fold, which resulted in only a 1.7 to 2.3-fold increase in the number of cleavage bonds identified in each donor sample (Figure S3). The NE-depleted donor samples shared 76 cleavage sites and 12 to 16 unique cleaved bonds were identified for each donor (Figure 5). The substrate signature of the shared cleavage sites showed a preference for phenylalanine, arginine and lysine in the P1 position, while arginine and norleucine were most often found in P3 and P2 sites, respectively. When compared to the individual serine protease datasets, we determined that 36 of the 76 cleavage sites were attributable to CG activity. In addition, PR3 and NSP4 accounted for 7 and 1 cleaved bonds, respectively, while the protease(s) responsible for 15 cleavage sites could not be determined. The successful depletion of NE was evident by the presence of a single NE cleaved bond, however, it is possible that cleavage at this site is due to an unidentified low abundance protease with overlapping specificity. In addition, hydrolysis of the PHWQRVIFFRLNTP peptide at the V-I site was not evident in the NE-depleted samples indicating that the cleavage site was the product of NE activity.

Taken together, we have utilized a novel substrate based profiling method to determine that NE and to a lesser extent CG and PR3 are active in neutrophil derived NETs. In addition, by selectively removing NE we have been able to perform an in-depth characterization of less abundant proteolytic activity and determined that NSP4 and at least one other protease are also active in NETs.

## Discussion

The formation of neutrophil extracellular traps is a novel host defense strategy in addition to the well characterized phagocytosis of neutrophils. Many groups have shown that viruses [20], bacteria [8] and fungi [21,22] stimulate neutrophils to release extracellular DNA, although it has been suggested that this process is an incidental component of neutrophil lysis or a hijacking of host pathways by the pathogen [23]. However, there is increasing evidence that NETosis is a highly orchestrated mechanism of cellular defense and is under tight control [24]. Irrespective of the mechanism by which chromatin and other cytotoxic components are released from neutrophils and assembled into NETs, the resultant damage to host tissue has been shown to promote inflammatory diseases such as cystic fibrosis [25], asthma [26], systemic vasculitis [7], systemic lupus erythematosus [27,28] and other acute and chronic diseases [9,29]. In unstimulated neutrophils, a set of homologous serine proteases are stored in azurophilic granules and their enzymatic activities are subject to intracellular compartmentalization and endogenous inhibitors [30]. Upon stimulation with PMA or bacteria they are released and selectively associated with extracellular DNA [14,31]. As extracellular proteins, the activity of these enzymes may no longer be under the control of inhibitors and, therefore, unregulated proteolysis could be initiated and prolong an inflammatory response [32]. Alternatively, association of these proteases with NETs could provide a means to compartmentalize and minimize their destructive proteolytic activities. It is worth noting that serine proteases seem to be selectively targeted to NETs since caspases that are important components of inflammasomes, and matrix metalloproteases were not detected on NETs. One simple explanation is that NE, CG and PR3 are positively charged basic proteins which have high affinity to the negatively charged DNA. Previous studies by Dubois and colleagues have used highly selective fluorescent substrates of NE, PR3 and CG to assess the proteolytic activity in NETs from Cystic Fibrosis sputum and from neutrophils stimulated with *S. aureus* and *P. aeruginosa* [25]. DNase treatment of sputum dramatically increased its NE activity, but not the activities of CG and PR3. They also determined that nuclease treatment of extracellular DNA from stimulated neutrophils caused a ~2.5 fold increase in soluble proteolytic activity relative to unstimulated cells, and that the contribution of each enzyme to the overall activity was approximately 40% for NE, 40% for PR3 and 20% for CG. Their study demonstrated that NETs are enriched in serine proteases and NE and PR3 are main contributors to NETs associated proteolytic activity.

In our study, we utilized a highly sensitive and unbiased substrate profiling method to characterize the entire proteolytic signature associated with NETs. MSP-MS is an ideal technology to profile complex biological samples because the substrate population consists of a defined set of peptides and, therefore, cleavage sites can be directly linked to a specific enzyme. Furthermore, the unbiased design of the peptides and combination into equimolar pools ensures that proteolytic activity from multiple proteases can be monitored simultaneously without prior knowledge of the substrate specificity. The reproducibility of both the sample preparation and the MSP-MS assay was evident as many of the peptides cleaved in three independent donor samples in this study were identical. Any variation between donors could be due to differences in genetic background, or immune status of each individual. Inter-individual variability has recently been highlighted by Barrientos and coworkers, where NET-associated proteolytic activity between donors differed by as much as 9-fold [33].

In our study, all of the proteolytic activity released from PMA-activated neutrophils could be attributed to NE, CG and PR3 activity. However, ~70% of cleavage sites were likely to be the result of NE activity and CG and PR3 contributed to the remaining 25% and 5%, respectively. As expected, when NE was immunodepleted from the NET extracts, CG became the dominant source of proteolytic activity. The remaining proteolytic activity was likely to be the product of PR3 and to a lesser extent NSP4 and other as yet unidentified NET associated proteases. Interestingly, PMA stimulation of neutrophils resulted in PR3 activity that was considerably lower than CG while other studies using bacterial stimulated neutrophils show the reverse. These data indicate that protease composition in NETs may differ depending on the type of stimulation. Khandpur and coworkers analyzed the composition in NETs following alternate stimulation and found differences in both the protein levels and protein composition [33,34].

Proteomic analysis was used to identify proteins released from the NETs, many of which have been observed in a previous study [14]. Interestingly, we found that NE is tightly bound to DNA and was not found in samples that lacked nuclease treatment. CG was found in all samples independent of treatment regimes while PR3 and NSP4 were not detected. Since robust PR3 activity was evident in the MSP-MS assay these data indicate that a functional activity assay has superior sensitivity than proteomic based methods for detecting proteases.

Previously, Proteomic Identification of Protease Cleavage Sites (PICS) was utilized to generate extended substrate specificity profiles of NE, CG and NSP4 [15,35]. In these studies, proteome derived peptides were used as the substrate library and cleavage by the neutrophil serine proteases was monitored by mass spectrometry. As was observed for the PS-SCL method, the substrate profiles using PICS strongly correlated with our MSP-MS data (Table S1). However, our study is the first to directly compare the substrate specificity of all four homologous neutrophil serine proteases under identical assay and instrumentation conditions, and reveals distinct cleavage sites that are uniquely cut by each enzyme. Traditionally, the neutrophil serine proteases were considered to have redundant functionalities based on their ability to hydrolyze structural proteins such as collagen, laminin, fibronectin and elastin [30]. However, out of the 180 bonds that were hydrolyzed by the four proteases only 28 cleavage sites were shared between two or more enzymes. Therefore, although our substrate profiling was performed using a synthetic peptide library, it is likely that many *in vivo* protein substrate cleavage sites exist that are unique to each neutrophil protease.

Successful substrate prediction tools require an experimentally determined list of protein or peptide substrates to identify sequence and structural features important for protease recognition. The predictive power for identifying natural substrates can be improved by incorporating negative substrates. Using the MSP-MS assay, any peptide bond that is cleaved by a neutrophil serine protease is a true substrate while every bond that is not cleaved is considered a true negative substrate. Using data generated in this study it may be possible to identify and track endogenous substrates of these proteases during neutrophil lysis and/or NET formation which will provide us with a greater understanding of roles of these enzymes in immune response and inflammation.

In this study, it is evident that targeting of NE on NETs could minimize adverse effects of unregulated proteolysis associated with tissue damage and inflammation. The selective cleavage sequences of the neutrophil serine proteases identified in this study will be valuable for designing substrates, inhibitors, activity based imaging agents [36] and protease-activated prodrugs [37]. In addition, the substrate signature of NETs-associated protease activity can be monitored as a biomarker for inflammatory diseases driven by extracellular neutrophil proteases.

## Materials and Methods

### Ethics statement

All data were analyzed anonymously and all aspects of this study were conducted according to the principles expressed in the Declaration of Helsinki. Human subject research approval for this study was received from the Western Institutional Review Board (study No: 1084207). All participants provided written informed consent prior to the start of the study.

### Isolation of neutrophils from healthy donors

Human neutrophil cells were isolated by a two-step purification protocol using Red Blood Cell (RBC) sedimentation followed by removal of monocytes using Ficoll density gradient centrifugation. Briefly, 50 ml of fresh human whole blood was collected in a collection tube containing heparin. Blood was mixed with HetaSep (STEM cell, Cat^#^ 07906) at the ratio (5:1) to precipitate RBCs and platelets. Supernatants with enriched leukocytes and monocytes were layered on top of Ficoll-PAQUE PLUS (GE Healthcare, Cat^#^ 17-1440-03). After Ficoll gradient centrifugation, neutrophils were separated from monocytes in the supernatant and pelleted. Contaminating RBCs were further removed by repeated cell lysis using RBC lysis buffer (Miltenyi Biotec, Cat^#^ 130-094-183). By this method, 50-100 million neutrophils were isolated to greater than 98% purity, as confirmed by flow cytometry using CD66b antibodies (BD Pharmamingen, Cat# 555724).

### NETosis induction and NET preparation

Purified neutrophils were washed five times to remove plasma proteins, then seeded at a density of 1.7 x 10^6^ cells/ml in RPMI 1640 media supplemented with glutamine in a 10 mm culture plate. NETosis was induced *in vitro* by stimulating neutrophils with 50 nM phorbol-12-myristate-13-acetate (PMA, Sigma P8139) at 37°C in a 5% CO_2_ incubator. After induction for 3 hours, media was removed and plates were washed gently with warm media for three times. The induction time was selected for maximum protein release from NETs after PMA treatment. NETs were then digested by Micrococcal Nuclease (MNase) (20 U/ml) (Thermo Scientific, Cat^#^ 88216) for 10-40 minutes and released into media. The supernatant was subsequently centrifuged to remove cells and cellular debris. Supernatants from PMA-untreated neutrophils and PMA-treated neutrophils but without MNase digestion were prepared as negative controls. A fraction of each sample was treated with protease inhibitor cocktail to preserve the sample for proteomic analysis, whereas the remainder of the sample for protease activity screening was not treated with protease inhibitors. Progression of NETosis was monitored by measuring cell-free DNA using Sytox Orange (Life Technologies, S11368). DNA was quantified by relative fluorescence measurement with a SpectraMax M2 fluorometer (Molecular Devices) at a filter setting of 544 nm (ex)/ 590 nm (em) and calibrated by standard curve with DNA standard of known concentration. NETosis was also visually examined by confocal immunofluorescent microscopy. Neutrophils (5 x 10^5^ cells/ml) were seeded on poly-L-lysine coated cover slips and treated with or without 50 nM PMA. At different time points post-NETosis induction, cells were fixed with 4% paraformaldehyde, then permeablized and blocked with 10% FBS in phosphate buffered saline (PBS) with 0.05% Triton X-100. For histone staining, coverslips were incubated with a mouse anti-human core histone antibody (Millipore, Anti-histone Clone H11-4, MAB3422) followed a Tetramethyl Rhodamine Isothiocyanate (TRITC)-conjugated secondary antibody (Invitrogen Cat^#^ T2762). DNA was counterstained with Hoechst 33342 (AnaSpec Inc, Cat^#^ 83218). Coverslips were mounted onto glass slides using Prolong Gold mounting media (Invitrogen Cat^#^ P36930) before acquisition.

### Determination of neutrophil serine protease activity

Human neutrophil serine proteases were purchased from Athens Research & Technology. A set of internally quenched fluorescent substrates (40 µM each) were assayed with 20 nM of NE (Cat^#^ 16-14-051200), CG (Cat^#^ 16-14-030107) and PR3 (Cat^#^ 16-14-161820) in Dulbecco’s-PBS containing 0.01% Tween-20 at room temperature. Triplicate assays were performed in 96-well plates in a Spectra Max Gemini EM (Molecular Devices) using a λ_ex_ 328nm and λ_em_ 393 nm. Initial velocities (relative fluorescent units/s) were calculated using SoftMax Pro software (Molecular Devices). NET samples were prepared as described above from three donors with the following combinations: +PMA/+MNase, +PMA/-MNase, and –PMA/+MNase treatment. 2 µg/ml of each NET sample was assayed in triplicate with 40 µM of (K-Mca) PLGKQVEY(K-Dnp). Initial velocities were converted to µmole/min/mg.

### Peptide cleavage site identification by multiplex substrate profiling-mass spectrometry

Recombinant NSP4 was expressed and isolated from HEK 293 cells as described previously [15]. NE (1 nM), CG (5 nM), PR3 (2 nM) and NSP4 (25nM) were profiled using the MSP-MS assay in Dulbecco’s-PBS as described previously [17]. In addition, proteolytic activities in three PMA-induced and MNase-treated donor NET samples were determined using the MSP-MS assay. Control samples lacked PMA or MNase treatment and consisted of an equal mixture of total protein from each donor. All assays contained 0.4 µg/mL of donor protein and 500 nM of each peptide in a total reaction volume of 900 µl. Aliquots were removed after 15, 60, 240, and 1200 minutes and quenched with concentrated formic acid to a final pH of 2.5. Samples were desalted and analyzed by LC-MSMS peptide sequencing.

For LC-MS/MS, an LTQ-FT mass spectrometer (Thermo) equipped with a 10,000 psi system nanoACUITY (Waters) UPLC instrument was used for reversed phase chromatography with a C18 column (BEH130, 1.7 µm bead size, 100 µm x 100 mm). The LC was operated at 600 nL/min flow rate, and peptides were separated using a linear gradient over 42 min from 2% B to 30% B, with solvent A: 0.1% formic acid in water and solvent B: 0.1% formic acid in 70% acetonitrile. Survey scans were recorded over 350-1800 m/z range, and MS/MS was performed with CID fragmentation on the six most intense precursor ions. Mass spectrometry peak lists were generated using in-house software called PAVA, and data were searched using Protein Prospector software v. 5.10.0 [38]. Data was searched against a database containing the sequences of the 124 14-mer synthetic peptides, concatenated with 4 different copies of randomized sequences for the same 124 entries to create a final database of 620 sequences for estimation of false discovery rate [39]. For database searching, peptide sequences were matched with no enzyme specificity requirement, and variable modifications including oxidation of Trp, Pro and Phe, and N-terminal pyroGlu from Gln. Protein Prospector score thresholds were selected to be minimum protein score of 20, minimum peptide score of 15, and maximum expectation values of 0.1 for “protein” and 0.05 for peptide matches, and resulted in a peptide false discovery rate of 0.2%. Cleavage site data was extracted from Protein Prospector using an in house script called “MSP extractor” software. The earliest time interval that ≥2.5% (n=41) of all possible bonds in the library (n = 1612) were cleaved was chosen to compare enzymes specificity. NE, PR3 and CG reached this value after 240, 1200 and 60 minutes incubation, respectively while NSP4 cleaved only 1.2% (n=19) of peptide bonds after 1200 minutes. For comparison of substrate specificity, an iceLogo software was used to generate substrate specificity logos for amino acids at ±4 positions adjacent to the identified cleavage sites [40].

### Protein identification of NETs by mass spectrometry

Protein identification in NET-induced samples was performed using peptide sequencing by mass spectrometry as previously reported [17]. The +PMA/+MNase treated samples were assayed individually for each donor, while the +PMA/-MNase, and -PMA/+MNase control samples were prepared as pooled mass-matched samples from the three donors. NET protein concentrations ranged from 25-40 µg/ml in PBS, therefore a slightly modified in solution trypsin digestion protocol was applied as follows. Samples were brought to a standardized concentration of 30 µg/ml with 100 mM ammonium bicarbonate buffer (~20 µg in a total volume of 700 µl) to which solid urea was added to 4M final. Sample was reduced with 10 mM DTT incubation for 10 min at 56 °C, then alkylated with 12 mM iodoacetamide (45 min, dark, 21 °C), and then quenched with 5 mM additional DTT. The final volume was adjusted to 1.4 ml with additional 100 mM ammonium bicarbonate, bringing urea concentration to 2M. Trypsin (sequencing grade, Promega) was added at 1:50 trypsin: total protein for digestion overnight at 37 °C. The sample was then acidified with formic acid to pH 2-3 and desalted using C18 OMIX tips (Varian). Each sample was assayed with two technical replicate LC-MS/MS analyses using an LTQ-Orbitrap (Thermo) mass spectrometer operated under identical separation and analysis conditions as the LTQ-FT system described above.

Database searches were performed against the *H. sapiens* UniProt database (downloaded March 21, 2012), containing 62,611 entries. For estimation of false discovery rate, this database was concatenated with a fully randomized set of sequence entries [36]. Data were searched with mass tolerances of 20 ppm for parent and 0.8 Da for fragment ions. Peptide sequences were matched as tryptic peptides with no missed cleavages, and carbamidomethylated cysteines as a fixed modification. Variable modifications included oxidation of Met, N-terminal pyroGlu from Gln, loss of Met and N-terminal acetylation. Protein Prospector score parameters were: minimum protein score of 22, minimum peptide score of 15, and maximum expectation values of 0.01 for protein and 0.001 for peptide matches, resulting in a protein false discovery rate of 1.1%. Protein identification results are reported with unique peptide count, peptide count as an approximation of protein abundance, percent sequence coverage and an expectation value for the probability of the protein identification [38,39]. Proteins were required to have been identified with at least two unique peptides in one of the three conditions tested (+PMA/+MNase, +PMA/-MNase, and -PMA/+MNase) to be reported.

### Proteolytic activity using combinatorial fluorogenic substrate libraries

Non-prime side sequence specificity, as the N-terminal sequence relative to the scissile bond is termed in protease nomenclature, was assayed for proteases using combinatorial fluorogenic substrate libraries [19]. This fluorescent peptide library is amenable for detailed profiling of purified serine proteases and distinguishes between subsite preferences in closely related enzymes. Human NE (50 nM), CG (100 nM), PR3 (50 nM) and NSP4 (100 nM) were assayed with this fluorogenic library in Dulbecco’s-PBS containing 0.01% Tween-20. Amino acid preferences at each position can be determined by direct comparison of activity, in units of picomolar of fluorophore released per second.

### Depletion of elastase from NET supernatants

MNase preparations of NET samples were secondarily digested with DNase (100U/ml) for 10 minutes at 37° to fully release NET-associated proteins. NET supernatants were then mixed with Pierce G/A magnetic beads (Thermo Scientific, Cat^#^ 88802) coated with elastase antibodies (Sigma, Cat^#^ PAI-74132) at 4 °C for 1 hour. After incubation, the supernatants were separated from beads on a DYNAL-magnet bead separation rack (Invitrogen Cat^#^ 123-21D).

## Supporting Information

Figure S1 **Screening of a set of internally quenched fluorescent peptides to identify a substrate that can be cleaved by NE (blue), PR3 (yellow) and CG (green).** All sequences have a N-terminal lysine(Mca) group and a C-terminal lysine(Dnp).
(TIF)

Figure S2 **Substrate specificity of human neutrophil serine proteases using the P4-P1 complete diverse positional scanning synthetic combinatorial library.** The *x* axis indicated the amino acids held constant at each position with “n” representing norleucine. All assays were performed in triplicate and the *y* axis indicates fluorescence released per second relative to the highest fluorescence observed for the enzyme at a single fixed position.
(TIF)

Figure S3 **Determination of the proteolytic signatures in NE-depleted NETs.** **A**-**C**. IceLogos representing the P4 to P4′ sites for NE-depleted NETs isolated from three donor samples. Amino acids that are most frequently observed (above axis) and least frequently observed (below axis) are illustrated. The numbers of cleavage sites used to make each iceLogo are listed in the bottom right-hand corner. Residues that are highlighted in black text are significantly (p = 0.05) enriched relative to the frequency that these same amino acids are found in the peptide library (5.2 +/- 0.5%). The amino acid ‘n’ corresponds to norleucine.
(TIF)

Table S1 **Comparison of NE, PR3 and NSP4 substrate specificity using MSP-MS and PICS.**
(DOCX)
