# Supplementary figures and images for: Global Substrate Profiling of Proteases in Human Neutrophil Extracellular Traps Reveals Consensus Motif Predominantly Contributed by Elastase
Source: PLoS One. 2013 Sep 20;8(9):e75141. doi: 10.1371/journal.pone.0075141 (PMC3779220; doi:10.1371/journal.pone.0075141)

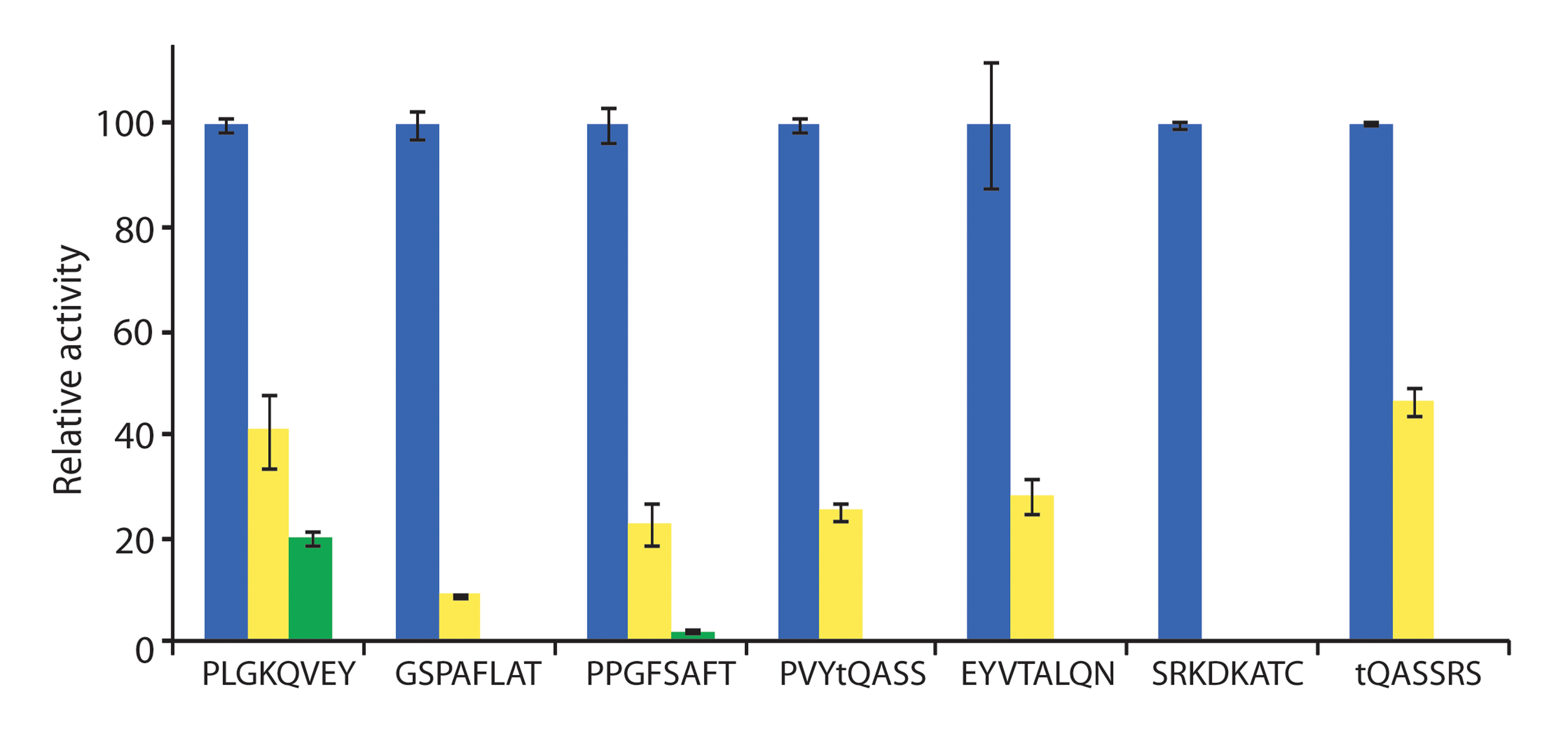

Supplement: Figure S1 — Screening of a set of internally quenched fluorescent peptides to identify a substrate that can be cleaved by NE (blue), PR3 (yellow) and CG (green). All sequences have a N-terminal lysine(Mca) group and a C-terminal lysine(Dnp). (TIF) [file pone.0075141.s001.tif]

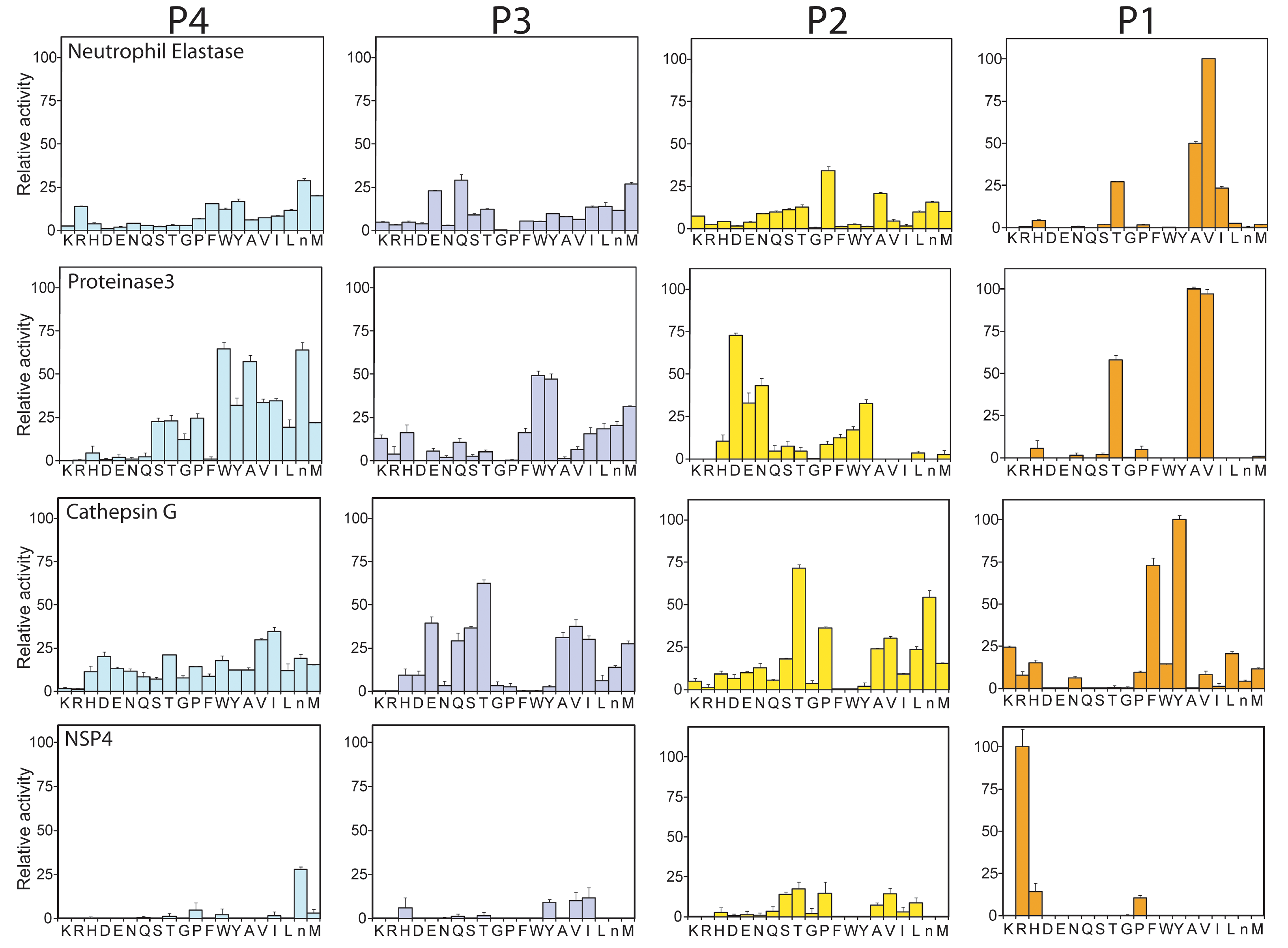

Supplement: Figure S2 — Substrate specificity of human neutrophil serine proteases using the P4-P1 complete diverse positional scanning synthetic combinatorial library. The x axis indicated the amino acids held constant at each position with “n” representing norleucine. All assays were performed in triplicate and the y axis indicates fluorescence released per second relative to the highest fluorescence observed for the enzyme at a single fixed position. (TIF) [file pone.0075141.s002.tif]

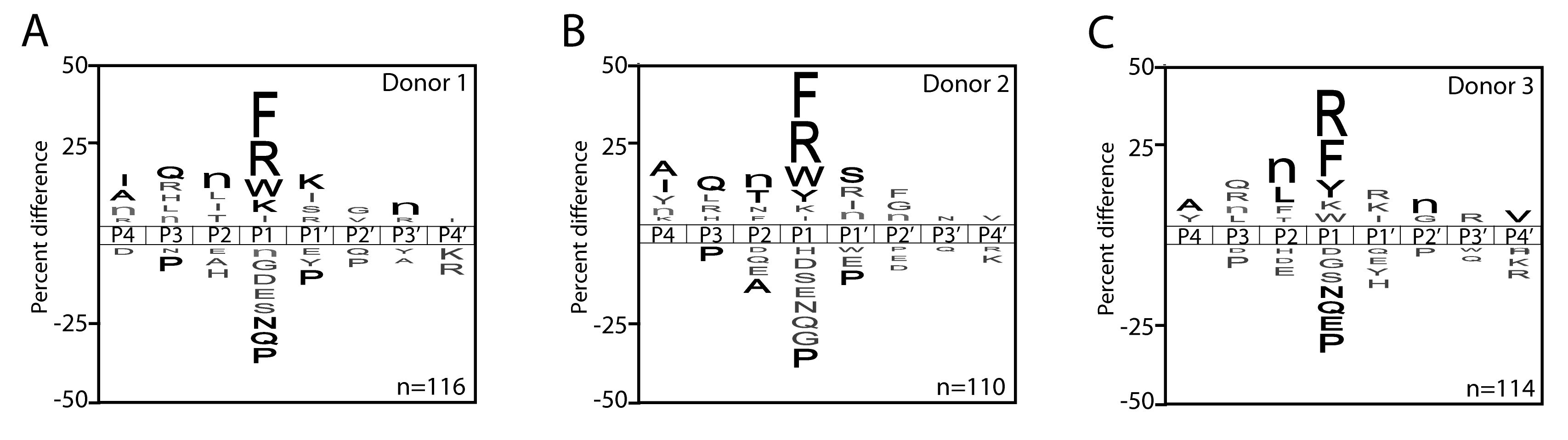

Supplement: Figure S3 — Determination of the proteolytic signatures in NE-depleted NETs. A-C. IceLogos representing the P4 to P4′ sites for NE-depleted NETs isolated from three donor samples. Amino acids that are most frequently observed (above axis) and least frequently observed (below axis) are illustrated. The numbers of cleavage sites used to make each iceLogo are listed in the bottom right-hand corner. Residues that are highlighted in black text are significantly (p = 0.05) enriched relative to the frequency that these same amino acids are found in the peptide library (5.2 +/- 0.5%). The amino acid ‘n’ corresponds to norleucine. (TIF) [file pone.0075141.s003.tif]
