# Supplementary material for: Global Substrate Profiling of Proteases in Human Neutrophil Extracellular Traps Reveals Consensus Motif Predominantly Contributed by Elastase
Source: PLoS One. 2013 Sep 20;8(9):e75141. doi: 10.1371/journal.pone.0075141 (PMC3779220; doi:10.1371/journal.pone.0075141)
Supplement: Table S1 — Comparison of NE, PR3 and NSP4 substrate specificity using MSP-MS and PICS. (DOCX) [file pone.0075141.s004.docx]

**Table S1 Comparison of NE, PR3 and NSP4 substrate specificity using MSP-MS and PICS**

|  | **P4** | **P3** | **P2** | **P1** | **P1'** | **P2'** | **P3'** | **P4'** |
| --- | --- | --- | --- | --- | --- | --- | --- | --- |
| NE | -0.15 | 0.53 | 0.29 | 0.87 | -0.03 | 0.46 | 0.01 | 0.05 |
| PR3 | -0.23 | 0.21 | 0.03 | 0.81 | 0.34 | 0.65 | 0.11 | 0.14 |
| NSP4 | 0.15 | 0.22 | -0.32 | 0.93 | 0.10 | 0.01 | -0.23 | -0.10 |

Values with no shading indicate weak or no correlation (Pearson) while grey and black represent strong and very strong correlation, respectively. CG has not been profiled using PICS so no correlation studies could be performed.
